# Association between antipsychotics and weight gain among psychiatric outpatients in Pakistan: a retrospective cohort study

**DOI:** 10.1186/1744-859X-7-12

**Published:** 2008-08-18

**Authors:** Syed Ahmer, Rashid AM Khan, Saleem Perwaiz Iqbal

**Affiliations:** 1 Department of Psychiatry, Aga Khan University, Karachi, Pakistan; 2 Department of Paediatrics & Child Health, Aga Khan University, Karachi, Pakistan

## Abstract

**Background:**

It has been known for a long time that use of antipsychotics, particularly atypical antipsychotics, is associated with weight gain and increase in risk of metabolic disturbances. In this study we have tried to find out if use of antipsychotics is associated with increase in weight and body mass index (BMI) in the Pakistani population.

**Methods:**

We performed a case note review of all patients who had been prescribed antipsychotic medication at the psychiatry outpatient clinic of a tertiary care university hospital in Pakistan over a 4-year period.

**Results:**

A total of 50% of patients had a BMI in the overweight or higher range at baseline. Patients showed a mean weight gain of 1.88 kg from baseline in 3 months and 3.29 kg in 6 months. Both of these values were statistically significant. The increase in mean BMI from baseline was 0.74 and 1.3 in 3 months and 6 months, respectively. In patients for whom we had at least one further weight measurement after baseline, 48% (39/81) showed a clinically significant weight gain.

**Conclusion:**

Pakistani patients are just as likely to put on weight during antipsychotic treatment as patients from other countries. Considering that this population already has a much higher prevalence of diabetes mellitus compared to the Western countries, the consequences of increased weight may be even more serious in terms of increased morbidity and mortality.

## Background

The mortality rate of people suffering from schizophrenia has been estimated to be twice as high as in the general population[1]. More than two thirds of this excess mortality is due to 'natural' causes[2], with death due to cardiovascular complications being the leading cause of this excess mortality[3].

The first reports of an increased risk of impaired glucose tolerance in people suffering from schizophrenia appeared in the literature several years before the first antipsychotic became available[4,5]. Soon after chlorpromazine was discovered reports suggesting an association between chlorpromazine and diabetes started appearing[6]. Since then many studies have been published firmly establishing a clear link between antipsychotics and diabetes mellitus, more with atypical than typical antipsychotics [7-10]. This led to a US Food and Drug Administration (FDA) recommendation in 2003 for including a warning about association with hyperglycaemia and diabetes on product labels for all atypical antipsychotics[9].

While it is not entirely clear how antipsychotics are linked to increased risk of impaired glucose tolerance and diabetes, weight gain and obesity are major side effects of many antipsychotics [11-13]. Obesity itself leads to hypertension, type II diabetes and coronary heart disease, many of the same problems that people with schizophrenia are already at an increased risk for[12].

We have not come across any research studying the association between antipsychotic use and weight gain in a Pakistani population. In this study, we have tried to find out if use of antipsychotics is associated with increase in weight and body mass index (BMI) in this population.

## Methods

The study was a case note review of all patients who had been prescribed antipsychotic medication in the psychiatry outpatient clinic of the Aga Khan University Hospital (AKUH) over a 4-year period. Patients were identified using the Psychiatric Assessment System (PAS), which records the basic demographic and clinical details including the medication prescribed, of patients presenting to the psychiatry clinics at the AKUH for the first time. All patients have their height recorded on the first visit and weight on every visit.

We calculated mean weight and BMI (weight in kg/height in m^2^) at baseline, 3 months and 6 months. A World Health Organization (WHO) expert consultation has suggested that the BMI cut-off points for determining overweight and obesity for Asian populations may be lower than Caucasian populations[14]. The consultation suggested the intervals of < 18.5, 18.5 to 23, 23 to 27.5 and ≥ 27.5, representing the categories of being underweight, increasing but acceptable risk, increased risk, and higher risk, respectively. We have used the same cut-offs in this study.

An increase in weight of 7% or more compared to the baseline is considered by licensing authorities as clinically significant weight gain[15]. We calculated how many patients had achieved clinically significant weight gain at 3 months and 6 months.

Statistical analyses were performed in SPSS v.15 (SPSS Inc., Chicago, IL, USA). We calculated means (with standard deviations) for quantitative variables and proportions (percentages) for categorical characteristics. We used a paired t test to determine if patients had achieved a statistically significant increase in weight and BMI from baseline. p Values < 0.05 were considered significant.

## Results

We found a total of 145 patients who had been seen at least once in the psychiatry clinic of AKUH and had been prescribed an antipsychotic medication. All of these had had their weight recorded at baseline. A total of 81 patients had at least 1 further weight measurement at least 3 months after the baseline measurement. In all, 33 patients had their weight measured at all 3 time points; baseline, 3 months and 6 months. A total of 56 people had been weighed at baseline and 3 months, and 60 people at baseline and 6 months.

The baseline sociodemographic and clinical characteristics of the sample are given in Table 1.

**Table 1:** Patient demographics and clinical characteristics at baseline

**Parameter**	**Value**
Age, years median (interquartile range)	31 (24–43)
Gender (n = 141):	
Male	79 (56%)
Female	62 (44%)
Marital status (n = 138):	
Single	75 (51%)
Married	52 (35.4%)
Widowed	7 (4.8%)
Divorced	3 (2%)
Separated	1 (0.7%)
Psychiatric diagnosis (n = 145):	
Schizophrenia	85 (57.8%)
Depression	21 14.3%
Bipolar disorder	16 (10.9%)
Delusional disorder	6 (4.1%)
Learning disability	5 (3.4%)
Dementia	3 (2%)
Substance misuse	3 (2%)
Obsessive/compulsive disorder (OCD)	2 (1.4%)
Anorexia nervosa	2 (1.4%)
Attention-deficit hyperactivity disorder (ADHD)	1 (0.7%)
Personality disorder	1 (0.7%)
Antipsychotic prescribed (n = 145):	
Risperidone	75 (51%)
Olanzapine	23 (15.6%)
Quetiapine	9 (6.1%)
Aripiprazole	3 (2%)
Clozapine	1 (0.7%)
Typical antipsychotics	34 (23.1%)

The mean weight and BMI of the total sample at baseline, 3 months and 6 months are shown in Table 2. Among all patients for whom we could calculate BMI (n = 140) 50% (70/140) had a BMI in the overweight or higher range (> 23) at baseline, 61% at 3 months and 63% at 6 months.

**Table 2:** Mean (SD) weight and body mass index (BMI)

	**Baseline**	**3 months**	**6 months**
Weight, kg	63.28 (16.99)	65.40 (18.01)	65.79 (15.79)
BMI, kg/m^2^	23.65 (5.45)	25.02 (5.48)	25.18 (4.93)

SD, standard deviation.

Patients for whom we had weight readings at baseline and 3 months (n = 56) showed a mean weight gain of 1.88 kilograms (63.51 vs 65.4 kg). This difference was statistically significant (t = -3.16, p value = 0.003). Patients for whom we had weight readings at baseline and 6 months (n = 60) showed a mean weight gain of 3.29 kilograms (62.5 vs 65.79 kg). This difference was also statistically significant (t = -2.95, p value = 0.004).

The difference in mean BMI at baseline and 3 months was 0.74 (24.27 and 25.02 respectively), which was statistically significant (p = 0.002). The difference in mean BMI between baseline and 6 months was 1.3 (23.84 and 25.18 respectively) and this increase was also statistically significant (p value = 0.002)

In patients for whom we had at least 1 further weight measurement after baseline, 48% (39/81) showed a clinically significant weight gain. In all, 51% (19/37) of patients on risperidone, 71% (8/11) on olanzapine and 16% (1/6) on quetiapine achieved clinically significant weight gain. However, the numbers were too small to meaningfully assess differences in the propensity of different antipsychotics to cause clinically significant weight gain.

We did a secondary analysis, dividing patients into groups by psychotic disorders, (schizophrenia, delusional disorder, drug-induced psychosis) and non-psychotic disorders (all other diagnoses) but the differences between the weights of these groups were non-significant at all time points (p value 0.671 at baseline, 0.238 at 3 months and 0.645 at 6 months).

A total of 91 patients were taking other psychotropic(s) besides an antipsychotic medication; 34 of these were taking SSRIs, 7 TCAs, 17 anticholinergics, 25 mood stabilisers (out of these 13 were taking valproic acid), 12 benzodiazepines, and 8 zolpidem. In all, 12 patients were taking other antidepressants including Mirtazapine (3), venlafaxine (5), and Mianserin (4).

## Discussion

In this study we found that almost 50% of patients had a BMI in the overweight or higher range according to the WHO suggested cut-offs for Asian populations at the start of the study. On average patients gained about 2 kg and 3.5 kg in weight from baseline in 3 and 6 months, respectively. This correlated with a BMI increase of 0.74 in 3 months and 1.3 in 6 months. About 48% of patients for whom we had at least 1 more weight reading after 3 or 6 months achieved a clinically significant weight gain.

In the study by Zipursky *et al. *[11] patients receiving olanzapine or haloperidol had a mean weight gain of 15.4 kg and 7.5 kg respectively. Allison *et al. *[12] in their systematic review reported a range of weight gain from 0.04 kg for ziprasidone to 4.45 kg for clozapine. Taylor and McAskill [13] concluded that all atypical antipsychotics, with the exception of ziprasidone (aripiprazole had not been marketed in 2000), have been associated with weight increases, with clozapine having the highest risk. The weight gain in our study was closer to the Allison than the Zipursky study. The main reason for this difference could be that in the Zipursky study patients were not recruited if they had received prior antipsychotic treatment for more than 16 cumulative weeks.

The overall prevalence of diabetes mellitus in Pakistan has been reported to be between 8.6% and 13.9%, depending on the province of residence [16-18]. This is far higher than the prevalence of diabetes of 1.2 to 6.3% reported from the US [8] or around 3% reported from the UK [19]. Any drug that causes weight gain is, therefore, likely to have even more serious consequences in terms of morbidity and mortality for the Pakistani population.

One of the limitations of our study was that almost all the patients had already received one or more antipsychotics for variable lengths of time before they first presented to the clinic at the AKUH. That may explain whey the weight gain in our study was not as stark as the Zipursky study[11]. Another limitation of the study is that there was no control group of patients who were not taking antipsychotic medications. This would have shed some light on how much of the weight gain might be attributable to suffering from a psychiatric illness and how much to taking of antipsychotic medications.

## Conclusion

Antipsychotics are associated with statistically significant weight gain in the Pakistani population. This may be even more hazardous for this population as the prevalence of diabetes mellitus is already higher than many other countries. It is important that while initiating an antipsychotic medication in this patient population, psychiatrists should counsel patients about the risk of weight gain associated with antipsychotic use, the increased risk of morbidity and mortality associated with weight gain, and the lifestyle changes such as changes in dietary habits and regular exercise that the patients can adopt to counter that risk.

## Competing interests

The authors declare that they have no competing interests.

## Authors' contributions

SA carried out the literature review, wrote the protocol, and wrote the initial draft of the paper. RK performed data extraction and was responsible for data entry into SPSS. SPI wrote the statistical part of the protocol/paper and carried out the statistical analyses. All authors were responsible for drafting the final form of the paper and approved the manuscript.
